# Iterative assessment of a sports rehydration beverage containing a novel amino acid formula on water uptake kinetics

**DOI:** 10.1007/s00394-024-03325-x

**Published:** 2024-02-13

**Authors:** Mark P. Funnell, Loris A. Juett, Kirsty M. Reynolds, Drusus A. Johnson, Ruth M. James, Stephen A. Mears, Samuel N. Cheuvront, Robert W. Kenefick, Lewis J. James

**Affiliations:** 1 National Centre for Sport and Exercise Medicine, School of Sport, Exercise and Health Sciences, Loughborough University, Leicestershire, LE11 3TU UK; 2 Sport, Health and Performance Enhancement Research Centre, School of Science and Technology, Nottingham Trent University, Nottingham, NG11 8NS UK; 3 Entrinsic Bioscience, LLC, Norwood, MA 02062 USA; 4 Sports Science Synergy, LLC, Franklin, MA 02038 USA

**Keywords:** Rate of absorption, Rehydration, Deuterium, Recovery, Fluid balance

## Abstract

**Purpose:**

Rapid gastric emptying and intestinal absorption of beverages is essential for rapid rehydration, and certain amino acids (AA) may augment fluid delivery. Three sugar-free beverages, containing differing AA concentrations (AA + PZ), were assessed for fluid absorption kinetics against commercial sugar-free (PZ, GZ) and carbohydrate-containing (GTQ) beverages.

**Methods:**

Healthy individuals (*n* = 15–17 per study) completed three randomised trials. Three beverages (550–600 mL) were ingested in each study (Study 1: AA + PZ [17.51 g/L AA], PZ, GZ; Study 2: AA + PZ [6.96 g/L AA], PZ, GZ; Study 3: AA + PZ [3.48 g/L AA], PZ, GTQ), containing 3.000 g deuterium oxide (D_2_O). Blood samples were collected pre-, 2-min, 5-min, and every 5-min until 60-min post-ingestion to quantify maximal D_2_O enrichment (Cmax), time Cmax occurred (Tmax) and area under the curve (AUC).

**Results:**

Study 1: AUC (AA + PZ: 15,184 ± 3532 δ‰ vs. VSMOW; PZ: 17,328 ± 3153 δ‰ vs. VSMOW; GZ: 17,749 ± 4204 δ‰ vs. VSMOW; *P* ≤ 0.006) and Tmax (*P* ≤ 0.005) were lower for AA + PZ vs. PZ/GZ. Study 2: D_2_O enrichment characteristics were not different amongst beverages (*P* ≥ 0.338). Study 3: Cmax (AA + PZ: 440 ± 94 δ‰ vs. VSMOW; PZ: 429 ± 83 δ‰ vs. VSMOW; GTQ: 398 ± 81 δ‰ vs. VSMOW) was greater (*P* = 0.046) for AA + PZ than GTQ, with no other differences (*P* ≥ 0.106).

**Conclusion:**

The addition of small amounts of AA (3.48 g/L) to a sugar-free beverage increased fluid delivery to the circulation compared to a carbohydrate-based beverage, but greater amounts (17.51 g/L) delayed delivery.

**Supplementary Information:**

The online version contains supplementary material available at 10.1007/s00394-024-03325-x.

## Introduction

Rapid gastric emptying and intestinal absorption of ingested fluid is essential for quickly replacing fluid losses incurred during exercise, heat stress and illness [1]. The rate at which ingested fluids are available to replace fluid losses is dependent on the speed the fluid empties from the stomach (i.e. gastric emptying rate) and the rate of absorption at the site of the intestine [2]. The constituent solutes of a beverage significantly affect the gastric emptying rate and intestinal absorption, and thus, how quickly the fluid enters the circulation [1–3]. Rehydration beverages typically contain a mixture of electrolytes and carbohydrate [3] and are formulated to promote rapid gastric emptying and intestinal absorption, consequently facilitating delivery of fluids to the circulation as quickly as possible [4–6].

Beverages spiked with heavy water (deuterium oxide, D_2_O) provide an integrated measure of both gastric emptying and intestinal absorption of fluids [7]. Although D_2_O does not provide a quantitative value for the amount of water delivered into the vasculature at any given time point, the temporal accumulation and kinetics of D_2_O can be mathematically described allowing for a quantitative comparison of differences in fluid absorption amongst beverages [8–11].

Studies have demonstrated superior D_2_O absorption kinetics when ingesting dilute carbohydrate beverages (i.e. sports beverages; < 6% carbohydrate) compared to water [11, 12]. Although higher glucose-containing beverages (> 6% carbohydrate) slow gastric emptying when compared to water [8, 9, 12], the active co-transport of two sodium molecules for every glucose molecule accelerates water absorption along the small intestine when compared to passive water absorption [13]. Nevertheless, growing health concerns over sugar sweetened beverages [14] have led to the wide-spread sale and consumption of sugar-free alternatives.

Artificially sweetened sugar-free beverages maintain palatability, improve voluntary fluid consumption, and minimise dehydration as well as sugar-containing beverages [15]. However, in the absence of carbohydrate carriers, both artificially sweetened sugar-free beverages and water are absorbed more slowly across the intestine than dilute sugar-containing beverages [11]. The inclusion of artificial non-nutritive sweeteners has no impact on fluid absorption [16], and therefore, artificially sweetened sugar-free beverages may be sub-optimal in scenarios where rapid delivery of ingested fluid into the circulation is required. At least when compared to low-concentration glucose beverages [11, 12].

One alternative to the inclusion of carbohydrate within rehydration beverages is the use of amino acids. The small intestine has the capacity for large-scale absorption of amino acids, dipeptides, and tripeptides, which can enhance the absorption of sodium and water across the small intestine [3, 17]. However, the selection and concentration of amino acids are important for a few reasons; (1) sodium stoichiometry varies amongst amino acids; (2) certain amino acids compete for the same intestinal transporters; (3) the density of amino acid transporters varies along the length of the small intestine; and (4) transporter saturation kinetics vary by amino acid [18–21]. Consequently, the inclusion of strategically selected amino acids into artificially sweetened sugar-free beverages may augment water absorption across the intestine [18], facilitating delivery to the circulation.

Therefore, the aim of the present study was to investigate the effect of the addition of differing amounts of a novel six-amino acid formula to a commercially available sugar-free rehydration beverage on water absorption, assessed via gastrointestinal D_2_O kinetics, and subsequent fluid balance markers. It was hypothesised that the addition of select amino acids would increase the rate of absorption of the sugar-free beverage, and the greater the amount of novel amino acid formula, the quicker the fluid absorption.

## Methods

### Design of studies

Three sequential studies assessing sugar-free rehydration beverages containing differing amounts of a novel amino acid formula were conducted. All studies received ethical approval from the Loughborough University Ethics Approvals (Human Participants) Sub-Committee (Study 1 ID: LEON3151; Study 2 ID: LEON1416; Study 3 ID: LEON3151-2859) and were registered with Clinical Trials (clinicaltrials.gov; Study 1 ID: NCT04819334; Study 2 ID: NCT04509388; Study 3 ID: NCT05698849). For Study 1 and 2, the amino acid beverages were compared to two commercially available sugar-free beverages, Powerade Zero™(PZ) and Gatorade Zero™ (GZ). For Study 3, the novel amino acid beverage was compared to a commercially available sugar-free rehydration beverage (PZ) and a commercially available 6% carbohydrate–electrolyte beverage (Gatorade Thirst Quencher™; GTQ). In all three studies, subjects completed a screening visit, and three experimental trials commencing at the same time of day (standardised within subjects between 08:00 and 09:00) in a randomised order, separated by ≥ 6 days.

### Screening visit

Before commencement of each study, subjects provided written informed consent, consent to publish, and completed a medical screening questionnaire. Subjects were healthy (according to a medical screening questionnaire), non-smokers and had no known history of cardiovascular, metabolic, digestive, or renal disease. During the screening visit, body mass (AFW-120 K, Adam Equipment Co., Milton Keynes, UK) and height (Seca 216, Hamburg, Germany) were measured, whilst body fat was estimated using skinfold measurements (Harpenden Skinfold Caliper, HaB International Ltd., Southam, UK) at the biceps, triceps, sub-scapula and supra-iliac [22], and subjects self-reported their activity levels. All skinfold measurements were taken by the same accredited The International Society for the Advancement of Kinanthropometry (ISAK) anthropometrist. The subject characteristics for the three studies are displayed in Table 1. There was no control for menstrual cycle phase as ovarian hormones/menstrual cycle phase do not appear to affect gastric emptying [23] or hydration outcomes [24].

**Table 1:** Subject characteristics for Study 1, 2 and 3 (mean ± SD)

	Study 1			Study 2			Study 3		
	Males	Females	Group mean	Males	Females	Group mean	Males	Females	Group mean
Subject number	10	5	15	10	7	17	12	3	15
Age (y)	27 ± 4	25 ± 1	27 ± 3	27 ± 3	25 ± 1	26 ± 3	29 ± 5	27 ± 3	28 ± 4
Height (m)	1.81 ± 0.08	1.63 ± 0.06	1.75 ± 0.11	1.83 ± 0.08	1.64 ± 0.06	1.75 ± 0.12	1.79 ± 0.07	1.64 ± 0.10	1.76 ± 0.09
Body mass (kg)	77.0 ± 12.2	54.8 ± 4.7	69.6 ± 14.8	80.2 ± 12.0	59.8 ± 8.0	71.8 ± 14.5	81.2 ± 11.1	56.3 ± 5.2	76.2 ± 14.4
BMI (kg/m^2^)	23.5 ± 3.0	20.7 ± 1.7	22.6 ± 3.0	23.8 ± 2.8	22.3 ± 3.0	23.2 ± 2.9	25.2 ± 2.5	20.9 ± 0.7	24.4 ± 2.9
Sum of 4-site skinfolds (mm)	29.7 ± 9.2	36.8 ± 10.1	32.1 ± 9.8	36.1 ± 15.1	48.6 ± 17.8	41.3 ± 16.9	38.6 ± 18.4	36.5 ± 10.8	38.2 ± 16.9
Estimated body fat (%)	12.2 ± 3.5	14.8 ± 3.4	13.0 ± 3.6	14.0 ± 5.0	17.8 ± 4.8	15.6 ± 5.1	14.7 ± 5.0	14.7 ± 3.8	14.7 ± 4.7
Activity level									
Training sessions per week	4 ± 1	4 ± 3	4 ± 2	5 ± 1	4 ± 3	5 ± 2	4 ± 1	5 ± 3	4 ± 2
Training volume (h/week)	4 ± 2	5 ± 6	4 ± 3	6 ± 2	5 ± 3	6 ± 2	5 ± 2	5 ± 4	5 ± 2

Sum of 4-site skinfolds (mm) = biceps, triceps, sub-scapular and supra-iliac

### Pre-trial standardisation

In each study, subjects completed a diet and physical activity record for the 24 h preceding their first experimental trial and replicated these patterns before the second and third experimental trials. Adherence was verbally checked on arrival for trials. Strenuous exercise or alcohol intake were not permitted during this period. The day before trials, subjects were instructed to consume a minimum of 40 mL/kg body mass of fluid [25, 26]. This volume included any fluid, i.e. water, juice, coffee, tea, carbonated drinks, etc. Subjects stopped eating and drinking at least 10 h before arrival at the laboratory.

### Experimental trials

Upon arrival at the laboratory, subjects voided their bladder into a plastic container, before nude body mass was recorded, and a flexible 20-gauge cannula was inserted into an antecubital/forearm vein for subsequent blood sampling. Subjects sat on a treatment bed with their legs flat on the bed and the backrest raised at ~ 55° (i.e. a semi-upright Fowler’s position). After 30 min, a baseline blood sample was taken. All blood samples were ~ 7.5 mL, and immediately, following each sample, the cannula was flushed with ~ 7.5 mL isotonic sterile saline (BD Biosciences, New Jersey, USA). A 550 mL (Study 1) or 500 mL bolus (Study 2 and 3) of the experimental beverage was then given, containing 3.000 g of deuterated water (Deuterium Oxide 99.9 atom % D, Sigma-Aldrich, St. Louis, USA), followed by a further 50 mL of the experimental beverage, which was used to swill around the drink vessel to ensure all deuterium oxide was ingested. Subjects were instructed to consume the beverage as quickly as possible, but to prioritise not spilling any. Subjects remained on the treatment bed in the semi-upright Fowler’s position for a further 60 min; the timer began at the commencement of drinking. Additional ~ 7.5 mL blood samples were taken at 2, 5, 10, 15, 20, 25, 30, 35, 40, 45, 50, 55 and 60 min. A second urine sample was collected after the final blood sample. Ambient temperature and relative humidity (Kestrel 4400, Nielsen-Kellerman Co., Philadelphia, USA) were recorded at 0, 30 and 60 min.

### Experimental beverages and blinding

Experimental beverages were administered in a double-blind manner, prepared by an investigator not involved in the data collection or analysis, and served in an opaque bottle. The composition of beverages for the three studies is detailed in Table 2. Protein was calculated from the sum of elemental amino acid gram weights (molecular weight x mM), which included in descending order by concentration, aspartic acid, serine, valine, isoleucine, threonine, and tyrosine. The proprietary amino acid ratios were held constant when adjusting mM and beverage gram weights up or down. The energy density was also estimated from the energy equivalent for whole proteins, allowing for small errors [27].

**Table 2:** Composition of sugar-free rehydration beverages containing differing amounts of a novel amino acid formula (AA + PZ) and commercially available control beverages (PZ, GZ and GTQ) for Study 1, 2 and 3

	AA + PZ			Control Beverages		
	1	2	3	PZ	GZ	GTQ
Study inclusion	1	2	3	1, 2, 3	1, 2	3
Energy (kJ/L)	292	116	58	0	0	1002
Protein (g/L)	17.51	6.96	3.48	0	0	0
Carbohydrate (g/L)	0	0	0	0	0	60
Fat (g/L)	0	0	0	0	0	0
Sodium (mmol/L)	18 ± 1	19 ± 0	18 ± 1	18 ± 1	19 ± 1	19 ± 1
Potassium (mmol/L)	2 ± 0	2 ± 0	2 ± 0	2 ± 0	3 ± 0	3 ± 0
Chloride (mmol/L)	20 ± 1	19 ± 1	18 ± 1	19 ± 1	9 ± 0	12 ± 1
Osmolality (mOsm/kg H_2_O)	209 ± 2	109 ± 2	87 ± 3	61 ± 3	49 ± 1	343 ± 33

AA + PZ = a sugar-free rehydration beverage (PZ) containing differing amounts of a novel amino acid formula. PZ = Powerade Zero™. GZ = Gatorade Zero™. GTQ = Gatorade Thirst Quencher™. Energy and macronutrient composition obtained from manufacturer information. Protein and energy density equivalents calculated from the sum of elemental amino acid gram weights (i.e. not whole protein). Sodium and potassium concentrations were analysed via flame photometry (M410C Flame Photometer, Sherwood Ltd., Cambridge, UK). Chloride concentrations were analysed via a chloride meter (M926S Chloride Analyser, Sherwood Ltd., Cambridge, UK). Osmolality was analysed via freezing-point depression (Gonotec Osmomat 030 Cryoscopic Osmometer, Gonotec, Berlin, Germany)

### Sample analysis

From each ~ 7.5 mL blood sample, ~ 1 mL was dispensed into a tube containing K_2_ EDTA (1.75 mg/mL; Teklab, Durham, UK). This was used to determine haemoglobin concentration and haematocrit via the cyanmethemoglobin method and microcentrifugation, respectively. These values were used to estimate changes in plasma volume relative to baseline [28]. These data were collected, and plasma volume estimated, at 10 timepoints in Study 1 (0, 2, 5, 10, 15, 20, 25, 30, 45, 60 min), 14 timepoints in Study 2 (0, 2, 5, 10, 15, 20, 25, 30, 35, 40, 45, 50, 55, 60 min), and 5 timepoints in Study 3 (0, 5, 15, 30, 60 min). For consistency, plasma volume is displayed at 5 timepoints for each of the three studies (Fig. 3). The reduction of plasma volume data from 10 and 14 timepoints to 5 timepoints in Study 1 and 2, respectively, did not alter the statistical outcomes/findings. From the remaining ~ 6.5 mL of whole blood, ~ 5 mL was dispensed into a second tube containing K_2_ EDTA (1.6 mg/mL; Sarstedt AG & Co., Nümbrecht, Germany), and ~ 1.3 mL was dispensed into a tube containing lithium heparin (0.25 mg/mL; Sarstedt AG & Co., Nümbrecht, Germany). Plasma was separated from both tubes by centrifugation (2500 g, 20 min, 4 °C) and frozen (− 80 °C) for subsequent analysis. Plasma samples used for D_2_O enrichment analysis were stored in glass vials.

Freezing-point depression (Gonotec Osmomat 030 Cryoscopic Osmometer; Gonotec, Berlin, Germany) was used to determine the osmolality of plasma from lithium heparin tubes. Urine specific gravity of both baseline and 60 min urine samples was measured on the day of trials (PAL-10S, Digital Urine Specific Gravity Refractometer, Atago Co. Ltd., Tokyo, Japan).

Plasma D_2_O enrichment was determined in duplicate using the Europa Scientific ANCA-GSL sample preparation unit and 20–20 isotope ratio mass spectrometry (Sercon Ltd., Cheshire, UK). In brief, an appropriate sample volume was pipetted into Exetainer tubes and an insert vial containing 5% platinum on alumina was added. The tubes were sealed and subsequently filled with pure hydrogen. Samples were left for an equilibration period, during which the isotopes in the solution exchanged with the hydrogen gas in the headspace. A sample of the headspace gas was then analysed by continuous-flow isotope ratio mass spectrometry. The isotopic enrichment data are expressed as δ‰ against the international water standard Vienna Standard Mean Ocean Water (VSMOW). The CV of this measurement was 0.23%. Plasma D_2_O enrichment area under the curve (AUC_60_) was calculated, and the maximal plasma D_2_O enrichment concentration observed at any measured time point (Cmax) and the time Cmax occurred (Tmax) were derived [29].

Additional results are provided in the supplementary material for Study 1 and 2 (plasma amino acids [Supplementary Figs. 1 and 2], glucose [Supplementary Fig. 3], lactate [Supplementary Fig. 4], creatinine [Supplementary Fig. 5]), and Study 2 only (plasma sodium [Supplementary Fig. 6A] and potassium [Supplementary Fig. 6B], and urine D_2_O concentration). Plasma amino acid concentrations were determined at 0, 15, 30, 45 and 60 min using a Biochrom 30 + high-performance liquid chromatography ion exchange system (Biochrom, Cambourne, UK). Plasma glucose, lactate, and creatinine at 0, 15, 30, 45 and 60 min were determined via enzymatic colorimetric method (ABX Pentra C400, Horiba Medical, Northampton, UK). Plasma sodium and potassium were determined at 0, 2, 5, 10, 15, 20, 25, 30, 35, 40, 45, 50, 55 and 60 min via flame photometry (M410C Flame Photometer, Sherwood Ltd., Cambridge, UK). Urine D_2_O concentration was determined via the method described above for plasma D_2_O enrichment; pre-trial urine D_2_O enrichment was subtracted from post-trial urine D_2_O enrichment to nullify any remaining D_2_O in the body water pool from previous experimental visits.

### Statistical analysis

Data were initially checked for normality of distribution using a Shapiro–Wilk test. Data containing two factors (Trial*Time) were initially analysed using two-way repeated measures analysis of variance (ANOVA) (SPSS version 27, SPSS Inc., Illinois, USA). Data containing one factor were initially analysed using one-way repeated measures ANOVA (normally distributed data) or Friedman’s ANOVA (non-normally distributed data). Where the assumption of sphericity was violated, the degrees of freedom were corrected using the Greenhouse–Geisser estimate. Significant ANOVA interaction (two-way ANOVA) and main (one-way ANOVA) effects were followed-up by post hoc paired *t* tests for normally distributed data, and Wilcoxon signed-rank tests for non-normally distributed data. The Holm-Bonferroni correction was applied to post hoc tests to control the family-wise error rate. A a-priori sample size estimation was performed using the data of Hill et al. [30] and Jeukendrup et al. [11], an α of 0.05, and a statistical power of 0.80. It was estimated that 15 subjects would be required per study to reject the null hypothesis for D_2_O kinetic parameters (e.g. AUC). Statistical significance was accepted when *P* < 0.05. All data are displayed as mean ± SD.

## Results

### Trial conditions

No differences were present for ambient temperature or relative humidity between trials in each of the three studies (*P* ≥ 0.130; Table 3). There were no differences between trials for pre-trial body mass, urine specific gravity (Table 3) or plasma osmolality (*P* ≥ 0.168; Fig. 2A–C), indicating subjects were in a similar hydration state at the beginning of trials in each of the three studies.

**Table 3:** Mean ambient temperature, mean relative humidity, pre-trial body mass and pre-trial urine specific gravity for the three experimental trials for Study 1, 2 and 3

	AA + PZ	PZ	GZ	GTQ	*p* value (ANOVA)
Study 1	***17.51 g/L AA***				
Ambient temperature (°C)	23.2 ± 0.9	23.2 ± 0.9	23.3 ± 0.8	–	0.967
Relative humidity (%)	32.2 ± 6.2	34.4 ± 6.9	32.9 ± 6.3	–	0.938
Pre-trial body mass (kg)	68.3 ± 14.7	68.3 ± 14.8	68.5 ± 14.8	–	0.191
Pre-trial USG	1.022 ± 0.005	1.021 ± 0.006	1.022 ± 0.004	–	0.863
Study 2	***6.96 g/L AA***				
Ambient temperature (°C)	24.6 ± 0.6	24.6 ± 0.5	24.3 ± 0.7	–	0.751
Relative humidity (%)	49.2 ± 9.1	47.3 ± 8.7	49.5 ± 9.3	–	0.130
Pre-trial body mass (kg)	70.3 ± 14.2	70.5 ± 14.1	70.4 ± 14.0	–	0.509
Pre-trial USG	1.021 ± 0.006	1.021 ± 0.005	1.022 ± 0.005	–	0.985
Study 3	***3.48 g/L AA***				
Ambient temperature (°C)	23.0 ± 1.2	22.8 ± 0.9	–	22.8 ± 1.3	0.186
Relative humidity (%)	40.1 ± 14.1	44.8 ± 10.9	–	42.7 ± 13.9	0.442
Pre-trial body mass (kg)	75.6 ± 14.5	75.6 ± 14.5	–	75.5 ± 14.3	0.675
Pre-trial USG	1.019 ± 0.005	1.019 ± 0.005	–	1.020 ± 0.004	0.746

Bold italic indicates amino acid concentration of AA-PZ of that study

Data are mean ± SD. USG = urine specific gravity. AA = amino acids. AA + PZ = a sugar-free rehydration beverage (PZ) containing differing amounts of a novel amino acid formula. PZ = Powerade Zero™. GZ = Gatorade Zero™. GTQ = Gatorade Thirst Quencher™. Ambient temperature and relative humidity data are mean of 0, 30 and 60 min time-points collapsed together

### Plasma D_2_O enrichment

**Fig. 1:**
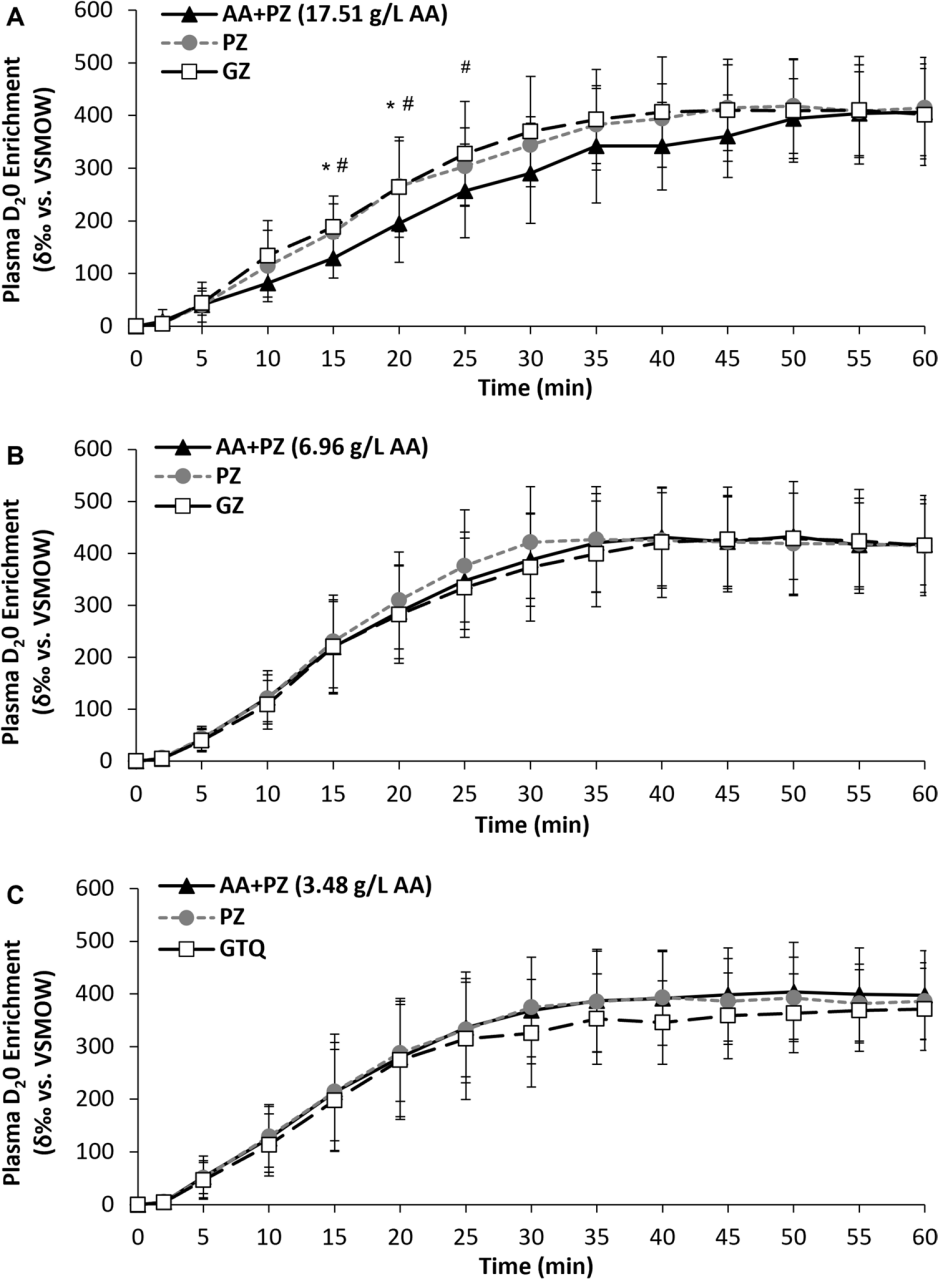
Plasma D_2_O enrichment (δ‰ vs. VSMOW) over time after ingesting the three expeirmental beverages for Study 1 (**A**), Study 2 (**B**) and Study 3 (**C**). * = AA + PZ significantly different to PZ. # = AA + PZ significnatly different to GZ. AA + PZ = a sugar-free rehydration beverage (PZ) containing differing amounts of a novel amino acid formula. PZ = Powerade Zero™. GZ = Gatorade Zero™. GTQ = Gatorade Thirst Quencher™

Study 1: There were main effects of time (*P* < 0.001), trial (*P* = 0.001), and a trial by time interaction effect (*P* < 0.001) for plasma D_2_O enrichment. Plasma D_2_O enrichment was lower at 15 and 20 min with AA + PZ compared to PZ (*P* ≤ 0.023), and lower at 15, 20 and 25 min with AA + PZ compared to GZ (*P* ≤ 0.006). There were no differences between PZ and GZ (*P* ≥ 0.240) for plasma D_2_O enrichment (Fig. 1A). There were main effects of trial for plasma D_2_O enrichment total AUC_60_ (*P* < 0.001) and Tmax (*P* = 0.005). Plasma D_2_O enrichment total AUC_60_ was lower with AA + PZ compared to PZ (*P* = 0.002) and GZ (*P* = 0.006), but there was no difference between PZ and GZ (*P* = 0.553). Tmax was greater with AA + PZ compared to PZ (*P* = 0.027) and GZ (*P* = 0.044), but there was no difference between PZ and GZ (*P* = 0.849). There was no difference in Cmax amongst trials (*P* = 0.612; Table 4).

**Table 4:** Plasma D_2_O enrichment characteristics for the three experimental beverages for Study 1, 2 and 3

	AA + PZ	PZ	GZ	GTQ	*p* value (ANOVA)
Study 1	***17.51 g/L AA***				
AUC_60_ (δ‰ vs. VSMOW)	15,184 ± 3532*	17,328 ± 3153	17,749 ± 4204	–	< 0.001
Cmax (δ‰ vs. VSMOW)	434 ± 91	447 ± 86	453 ± 110	–	0.612
Tmax (min)	51 ± 11*	45 ± 12	45 ± 12	–	0.005
Study 2	***6.96 g/L AA***				
AUC_60_ (δ‰ vs. VSMOW)	18,655 ± 3541	19,063 ± 4308	18,290 ± 4355	–	0.584
Cmax (δ‰ vs. VSMOW)	469 ± 80	476 ± 110	458 ± 104	–	0.573
Tmax (min)	41 ± 13	41 ± 11	45 ± 11	–	0.338
Study 3	***3.48 g/L AA***				
AUC_60_ (δ‰ vs. VSMOW)	17,731 ± 4101	17,564 ± 3732	–	16,195 ± 4209	0.114
Cmax (δ‰ vs. VSMOW)	440 ± 94^#^	429 ± 83	–	398 ± 81	0.030
Tmax (min)	45 ± 15	40 ± 14	–	45 ± 12	0.206

Bold italic indicates amino acid concentration of AA-PZ of that study

Data are mean ± SD. AUC_60_ = total area under the curve for 60 min. Cmax = maximal concentration of plasma D_2_O enrichment. Tmax = the time which Cmax occurred. AA = amino acids. AA + PZ = a sugar-free rehydration beverage (PZ) containing differing amounts of a novel amino acid formula. PZ = Powerade Zero™. GZ = Gatorade Zero™. GTQ = Gatorade Thirst Quencher™. * = AA + PZ significantly different to PZ and GZ. ^#^ = AA + PZ significantly different to GTQ

Study 2: There was a main effect of time (*P* < 0.001), with an initial increase in plasma D_2_O enrichment until ~ 35 min before reaching a plateau. There were no trial or trial by time interaction effects for plasma D_2_O enrichment (*P* ≥ 0.252; Fig. 1B). There were no differences in plasma D_2_O characteristics amongst trials (*P* ≥ 0.338; Table 4).

Study 3: There was a main effect of time (*P* < 0.001), with an initial increase in plasma D_2_O enrichment until ~ 35 min before reaching a plateau. There were no trial or trial by time interaction effects for plasma D_2_O enrichment (*P* ≥ 0.108; Fig. 1C). There were no differences amongst trials for total AUC_60_ or Tmax (*P* ≥ 0.114). There was a main effect of trial for Cmax (*P* = 0.030), which was greater with AA + PZ compared to GTQ (*P* = 0.046), but not PZ (*P* = 0.498). There was no difference in Cmax between GTQ and PZ (*P* = 0.106; Table 4).

### Plasma osmolality

**Fig. 2:**
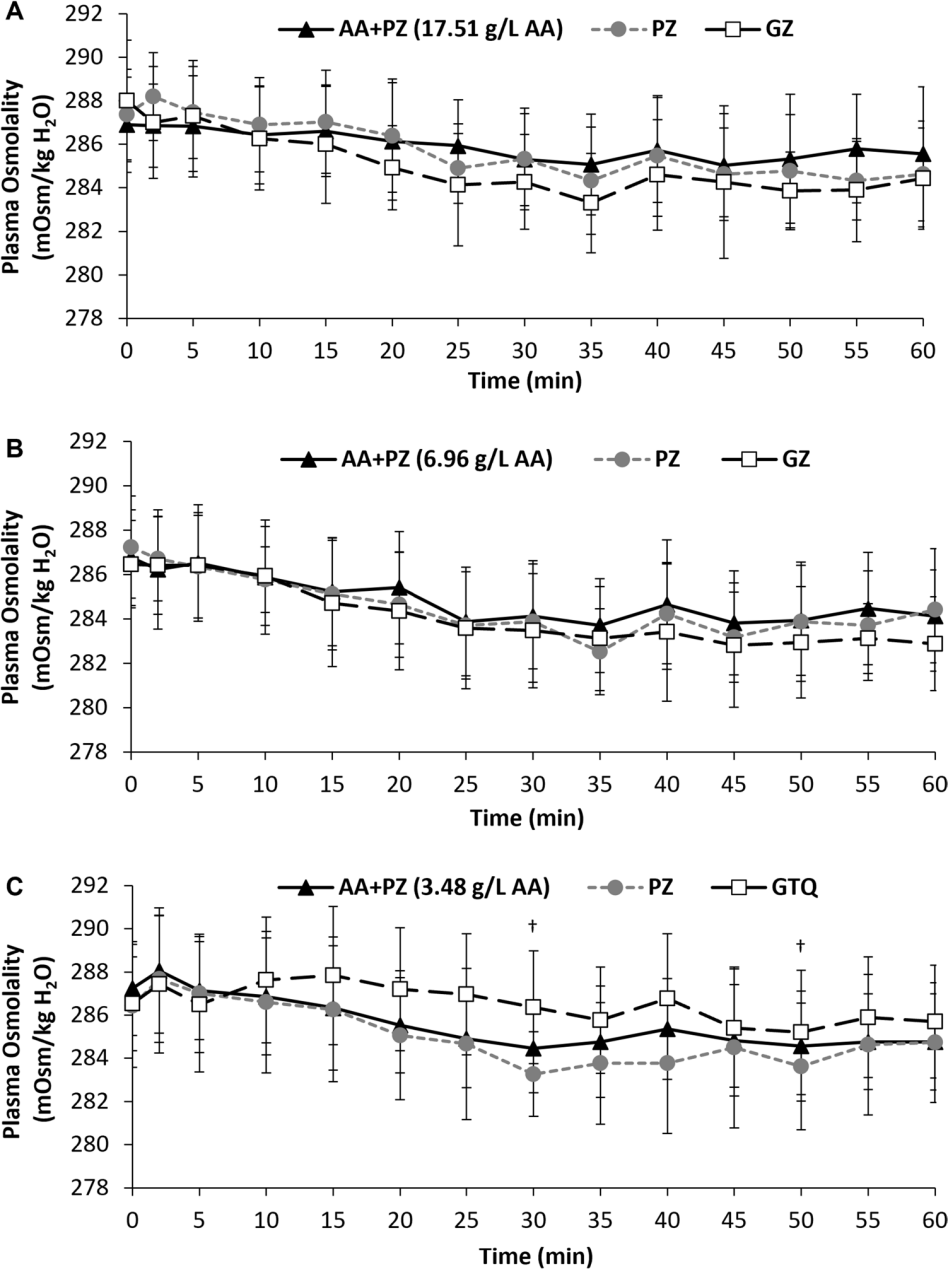
Plasma osmolality (mOsm/kg H_2_O) over time after ingesting the three experimental beverages for Study 1 (**A**), Study 2 (**B**) and Study 3 (**C**). † = GTQ significantly different from PZ. AA + PZ = a sugar-free rehydration beverage (PZ) containing differing amounts of a novel amino acid formula. PZ = Powerade Zero™. GZ = Gatorade Zero™. GTQ = Gatorade Thirst Quencher™

Study 1: There was a main effect of time (*P* < 0.001) for plasma osmolalility, with an initial decrease in plasma osmolality until ~ 35 min before reaching a plateau. There was a trial by time interaction effect (*P* < 0.001), but no effect of trial (*P* = 0.142) for plasma osmolality (Fig. 2A). Post hoc tests revealed no significant differences between trials after correction for multiple comparisons (*P* ≥ 0.137).

Study 2: There was a main effect of time (*P* < 0.001) for plasma osmolalility, with an initial decrease in plasma osmolality until ~ 35 min before reaching a plateau. There were no trial or trial by time interaction effects for plasma osmolality (*P* ≥ 0.228; Fig. 2B).

Study 3: There were main effects for time (*P* < 0.001), trial (*P* = 0.022), and a trial by time interaction effect (*P* = 0.007) for plasma osmolality (Fig. 2C). Plasma osmolality was greater (*P* ≤ 0.024) at 30 min and 50 min after consumption of GTQ compared to PZ, post hoc tests revealed no further differences between trials (*P* ≥ 0.076). After consumption of AA + PZ, plasma osmolality was not different from baseline for the first 15 min (*P* ≥ 0.153), but was significantly lower than baseline from 20 min onwards (*P* ≤ 0.032). After consumption of PZ, plasma osmolality was not different from baseline for the first 25 min (*P* ≥ 0.096), but was significantly lower than baseline from 30 min onwards (*P* ≤ 0.039). After consumption of GTQ, plasma osmolality was not different to baseline at any time point (*P* ≥ 0.192).

### Plasma volume

**Fig. 3:**
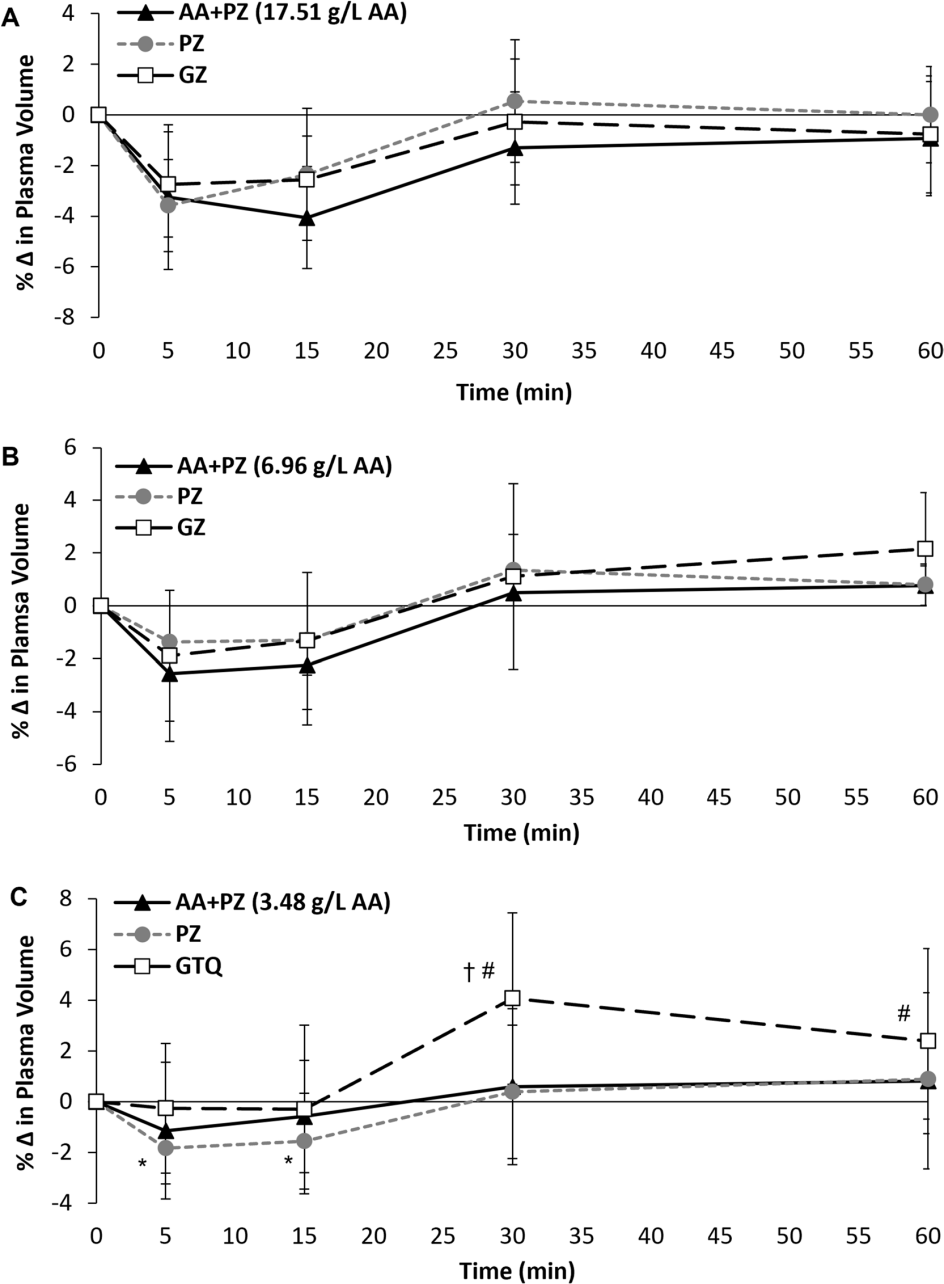
Change in plasma volume (%) relative to 0 min after ingesting the three experimental beverages for Study 1 (**A**), Study 2 (**B**) and Study 3 (**C**). † = GTQ significantly different from AA + PZ and PZ. * = time point significantly different from 0 min within PZ trial. # = time point significantly different from 0 min within GTQ trial. AA + PZ = a sugar-free rehydration beverage (PZ) containing differing amounts of a novel amino acid formula. PZ = Powerade Zero™. GZ = Gatorade Zero™. GTQ = Gatorade Thirst Quencher™

Study 1: There was a main effect of time (*P* < 0.001) for change in plasma volume; with plasma volume significantly lower than baseline at 5 and 15 min (*P* ≤ 0.001), but not different to baseline at 30 and 60 min (*P* ≥ 0.163). There were no trial (*P* = 0.102) or trial by time interaction effects (*P* = 0.124) for change in plasma volume (Fig. 3A).

Study 2: There was a main effect of time (*P* < 0.001) for change in plasma volume; with plasma volume initially decreasing below baseline at 5 and 15 min (*P* ≤ 0.001), before increasing above baseline at 30 and 60 min (*P* ≤ 0.029). There were no trial (*P* = 0.468) or time by trial interaction effects (*P* = 0.474) for change in plasma volume (Fig. 3B).

Study 3: There were main effects for time (*P* < 0.001), trial (*P* = 0.028) and a trial by time interaction effect (*P* = 0.002) for change in plasma volume (Fig. 3C). Plasma volume was greater at 30 min after consumption of GTQ compared to PZ and AA + PZ (*P* ≤ 0.045), with no further differences between trials (*P* ≥ 0.123). Plasma volume was not different from baseline at any time point after consumption of AA + PZ (*P* ≥ 0.074). After consumption of PZ, plasma volume was significantly lower than baseline at 5 and 15 min (*P* ≤ 0.019), but was not different to baseline at 30 and 60 min (*P* ≥ 0.176). After consumption of GTQ, plasma volume was not different to baseline at 5 and 15 min (*P* ≥ 0.470), but was greater than baseline at 30 and 60 min (*P* ≤ 0.036).

### Urine specific gravity and urine volume

No differences were present between trials for post-trial urine specific gravity in any of the three studies (*P* ≥ 0.406; Table 5). No differences were present between trials in Study 1 and 2 for post-trial urine volume (*P* ≥ 0.841). Post-trial urine volume was significantly different between trials in Study 3 (*P* = 0.033; Table 5), with lower volume after consumption of GTQ compared to PZ (*P* = 0.046), but not AA + PZ (*P* = 0.149). There was no difference in post-trial urine volume between AA + PZ and PZ (*P* > 0.999).

**Table 5:** Post-trial (60 min) urine volume and urine specific gravity for the three experimental beverages for Study 1, 2 and 3

	AA + PZ	PZ	GZ	GTQ	*p* value (ANOVA)
Study 1	***17.51 g/L AA***				
Urine volume (mL)	342 ± 109	355 ± 128	332 ± 98	–	0.841
USG	1.006 ± 0.003	1.005 ± 0.002	1.005 ± 0.002	–	0.676
Study 2	***6.96 g/L AA***				
Urine volume (mL)	251 ± 116	256 ± 125	260 ± 113	–	0.954
USG	1.009 ± 0.006	1.008 ± 0.004	1.009 ± 0.005	–	0.901
Study 3	***3.48 g/L AA***				
Urine volume (mL)	335 ± 134	336 ± 110	–	268 ± 79*	0.033
USG	1.005 ± 0.003	1.005 ± 0.002	–	1.006 ± 0.003	0.406

Bold italic indicates amino acid concentration of AA-PZ of that study

Data are mean ± SD. USG = urine specific gravity. AA = amino acids. AA + PZ = a sugar-free rehydration beverage (PZ) containing differing amounts of a novel amino acid formula. PZ = Powerade Zero™. GZ = Gatorade Zero™. GTQ = Gatorade Thirst Quencher™. * = GTQ significantly lower than PZ. * = GTQ significantly lower than PZ

## Discussion

Three studies were conducted to assess the addition of differing amounts of a novel amino acid formula to a sugar-free rehydration beverage on fluid absorption. Fluid absorption was assessed via gastrointestinal D_2_O kinetics, and the effect on subsequent fluid markers was measured. It was hypothesised that the rate of absorption of a sugar-free rehydration beverage would increase as the amount of amino acids added to the beverage increased. However, opposed to the hypothesis, the addition of greater amounts of the amino acid mixture (17.51 g/L) to a sugar-free rehydration beverage delayed water delivery to the circulation and neither lower concentration amino acid beverage demonstrated differences in water uptake kinetics compared to the sugar-free beverages. In contrast, when compared to a 6% carbohydrate beverage (GTQ), the addition of a smaller amount of amino acids (3.48 g/L) to a sugar-free rehydration beverage increased fluid delivery, evidenced by a greater maximal plasma D_2_O enrichment concentration.

Water-soluble organic molecules, which include certain amino acids, dipeptides and tripeptides, when absorbed from the small intestine enhance the absorption of electrolytes and water [20, 31–33]. The aim of Study 1 was to provide as many grams of the amino acid formula as possible within the sugar-free rehydration beverage; based upon solubility and flavour, 17.51 g/L of amino acid formula was delivered in a 600 mL bolus (i.e. a total of 10.5 g of amino acids). The delayed time of maximal plasma D_2_O enrichment concentration, and lower plasma D_2_O enrichment AUC, demonstrate a delay in water delivery into the circulation after consumption of the amino acid beverage compared to two commercially available sugar-free rehydration beverages.

The use of a D_2_O tracer is an integrated measure of gastric emptying and intestinal absorption. Therefore, the delay in D_2_O appearance in the circulation after consumption of the novel amino acid beverage was a result of delayed gastric emptying, and/or intestinal amino acid transporter saturation, or a combination of both [1, 20]. The greater energy density, and potentially osmolality, of the novel amino acid beverage may have delayed gastric emptying [1, 34], impeding fluid delivery to the proximal intestine and consequently circulation. Once emptied from the stomach, fluids delivered to the duodenum are quickly brought into osmotic equilibrium with the circulating plasma, and the jejunum is relatively permeable to electrolytes and water [1]. Therefore, although the initial beverage was hypotonic (209 ± 2 mOsm/kg H_2_O), if a portion of the water and electrolytes from the beverage were rapidly absorbed in the proximal intestine, a high concentration of amino acids would remain in the small intestine. This would raise the osmolality of the small intestine above that of the plasma [35], resulting in ‘osmotic backflow’ (fluid osmotically moving from the extracellular fluid to the intestinal lumen [20], potentially negating any beneficial effects of increased absorption induced by amino acid transport along the intestine [1, 2, 35]. Although there is limited data on beverage amino acid content and gastrointestinal D_2_O kinetics, similar inferior D_2_O absorption kinetics have been observed with higher glucose-containing beverages (> 6% carbohydrate) compared to more dilute carbohydrate beverages and water [8, 9, 11, 12, 35].

For Study 2, a smaller amount of amino acid formula (6.96 g/L) within the sugar-free rehydration beverage was delivered in a marginally smaller 550 mL bolus (i.e. a total of 3.83 g of amino acids). The addition of 6.96 g/L of amino acids into a rehydration beverage resulted in comparable water delivery into the circulation compared to two commercially available sugar-free rehydration beverages. Receptors in the duodenum and ilium are sensitive to macronutrient content, pH, and osmotic pressure [1, 36], and the activity of these receptors can delay gastric emptying by initiating hormonal and neural responses that alter gastric and duodenal muscular contraction [1]. Therefore, increasing macronutrient content and energy density of a beverage can delay gastric emptying [36, 37]. One hypothesis is that the inclusion of amino acids in the rehydration beverage may have delayed gastric emptying whilst concurrently increasing net sodium and water transport across the intestine [1]. Therefore, the beneficial effect of increased intestinal absorption may have been negated by a decreased gastric emptying rate. On the contrary, a second hypothesis is that, due to the relatively low amino acid content of the beverage, gastric emptying may have been similar between beverages, resulting in a comparable amount of fluid rapidly absorbed in the duodenum, and subsequently similar fluid delivery between beverages. However, with limited research on amino acid containing beverages and fluid delivery, it is difficult to fully ascertain.

Nevertheless, due to the delayed or equivalent water delivery of the novel amino acid beverages in Study 1 and 2, respectively, the amount of amino acid formula within the sugar-free rehydration beverage was further reduced to 3.48 g/L in a 550 mL bolus (i.e. a total of 1.91 g of amino acids). Additionally, the results from Study 1 and 2 demonstrate that both sugar-free rehydration beverages (PZ and GZ) have similar gastrointestinal D_2_O kinetics, a commercially available 6% carbohydrate–electrolyte beverage (GTQ) and a sugar-free rehydration beverage (PZ) were used for comparison in Study 3. The addition of a smaller amount of novel amino acid formula to a sugar-free rehydration beverage increased fluid delivery compared to the commercially available carbohydrate–electrolyte beverage, evidenced by greater maximal plasma D_2_O enrichment. However, there were no differences in D_2_O delivery into the circulation between the sugar-free rehydration beverage and amino acid beverage.

The small intestine has the capacity for fast and large-scale absorption of amino acids, dipeptides, and tripeptides, which can enhance the absorption of sodium and water across the small intestine [3, 17]. The low concentration of amino acids within the beverage likely emptied rapidly from the stomach and increased water and sodium transport across the intestine, resulting in greater water delivery compared to the carbohydrate–electrolyte beverage [18–20]. However, there may also have been inhibitory feedback from duodenal osmoreceptors and the glucose–sodium cotransporter (SGLT1) in the jejunal epithelium (to prevent the absorptive capacity of the proximal intestine becoming overwhelmed) that could have reduced gastric emptying after consumption of the carbohydrate–electrolyte beverage [12]. The greater maximal plasma D_2_O enrichment with the amino acid beverage, but not the sugar-free rehydration beverage, compared to the carbohydrate–electrolyte beverage suggests the amino acids may have marginally accelerated water delivery and warrants further investigation.

Plasma D_2_O enrichment does not reflect absorption per se, as it requires extra- and intra-cellular volumes to remain constant during the sampling period [11]. This was likely not the case, for example in Study 3, an expansion in plasma volume occurred 30–60 min after consumption of the 6% carbohydrate–electrolyte beverage. If plasma volume expanded, an increase in fluid absorption from the intestine could have occurred without a parallel increase in plasma D_2_O enrichment [11]. Due to potential alterations in extra- and intra-cellular volumes, the D_2_O uptake results should be treated with caution.

The lower post-trial urine output following consumption of the 6% carbohydrate–electrolyte beverage in Study 3 indicates greater beverage retention. Plasma osmolality influences circulating arginine vasopressin concentrations, and arginine vasopressin concentrations are responsible for the re-absorption of water in the kidney and thus urine production [35, 38]. Lessening urine production is pivotal in maximising rehydration beverage retention [39], and this occurs by minimising the reduction in plasma osmolality and associated circulating arginine vasopressin concentrations following beverage consumption [4, 40, 41]. Plasma osmolality did not differ from baseline after consumption of the carbohydrate–electrolyte beverage, whereas plasma osmolality decreased below baseline following consumption of the sugar-free rehydration beverage (with or without amino acids). Therefore, the greater plasma osmolality and expected greater associated circulating arginine vasopressin concentrations, after consumption of the 6% carbohydrate beverage was likely responsible for the lower urine output [41, 42]. Given the similarity in electrolyte composition of the beverages used across the studies, these effects are likely directly attributable to the differences in carbohydrate content [42]. Interestingly, previous studies [43, 44] suggest larger carbohydrate concentrations (> 10%) are required to decrease post-ingestion urine output, in contrast to the present findings. It could be that the lack of a difference in these previous studies is explained by the larger drink volume (1000 mL vs. 550 mL in Study 3) causing a volume-induced diuresis that masked the more subtle effects of lower carbohydrate contents.

The greater plasma osmolality and potentially decreased urine output, following consumption of the 6% carbohydrate–electrolyte beverage was likely responsible for the observed increase in plasma volume at 30 and 60 min. However, the greater plasma volume at 30 min post-consumption of the 6% carbohydrate–electrolyte beverage was unlikely the result of increased gastrointestinal absorption of the beverage as the D_2_O enrichment characteristics were not superior to the other beverages. Therefore, the increase in plasma volume likely derived from movement of interstitial or intracellular fluid augmented by the higher plasma osmolality, and not from the beverage itself.

## Conclusion

In conclusion, the addition of a small amount of a novel amino acid formula (3.48 g/L) to a sugar-free rehydration beverage increased water delivery into the circulation compared to a 6% carbohydrate-containing rehydration beverage. However, the addition of greater amounts of amino acids (17.51 g/L) to a sugar-free rehydration beverage delayed fluid delivery, potentially due to delayed gastric emptying and/or intestinal transporter saturation.

Future research should assess a sugar-free rehydration beverage containing a small amount of a novel amino acid formula in scenarios where rapid delivery of water into the circulation is required (i.e. illness, heat stress or exercise). The gastrointestinal water uptake kinetics of differing amalgams of amino acids, dipeptides, and tripeptides, within rehydration solutions, with the aim to further increase the rate of fluid delivery, should also be investigated. Additionally, age-related differences in fluid retention [42], potentially due to changes in renal function, and/or fluid absorption kinetics [45], mean it would be prudent to confirm the current findings in differing populations (i.e. older adults).

### Supplementary Information

Below is the link to the electronic supplementary material.

Supplementary file1 (DOCX 982 KB)

## Data Availability

Data is available upon reasonable request to the corresponding author.

## References

[1] Leiper JB (2015). Fate of ingested fluids: factors affecting gastric emptying and intestinal absorption of beverages in humans. Nutr Rev.

[2] Leiper JB (1998). Intestinal water absorption—implications for the formulation of rehydration solutions. Int J Sports Med.

[3] Bhan MK, Mahalanabis D, Fontaine O, Pierce NF (1994). Clinical trials of improved oral rehydration salt formulations: a review. Bull World Health Organ.

[4] Clayton DJ, Evans GH, James LJ (2014). Effect of drink carbohydrate content on postexercise gastric emptying, rehydration, and the calculation of net fluid balance. Int J Sport Nutr Exerc Metab.

[5] Shirreffs SM, Taylor AJ, Leiper JB, Maughan RJ (1996). Post-exercise rehydration in man: effects of volume consumed and drink sodium content. Med Sci Sports Exerc.

[6] Shirreffs SM, Aragon-Vargas LF, Keil M (2007). Rehydration after exercise in the heat: a comparison of 4 commonly used drinks. Int J Sport Nutr Exerc Metab.

[7] Lambert CP, Ball D, Leiper JB, Maughan RJ (1999). The use of a deuterium tracer technique to follow the fate of fluids ingested by human subjects: effects of drink volume and tracer concentration and content. Exp Physiol.

[8] Murray R, Bartoli W, Eddy D, Horn M (1997). Gastric emptying and plasma deuterium accumulation following ingestion of water and two carbohydrate-electrolyte beverages. Int J Sport Nutr.

[9] Davis JM, Lamb DR, Burgess WA, Bartoli WP (1987). Accumulation of deuterium oxide in body fluids after ingestion of D2O-labeled beverages. J Appl Physiol.

[10] Péronnet F, Mignault D, Du Souich P (2012). Pharmacokinetic analysis of absorption, distribution and disappearance of ingested water labeled with D2O in humans. Eur J Appl Physiol.

[11] Jeukendrup AE, Currell K, Clarke J (2009). Effect of beverage glucose and sodium content on fluid delivery. Nutr Metab (Lond).

[12] Shi X, Osterberg KL, Petrie H (2017). Effect of different osmolalities, CHO types, and [CHO] on gastric emptying in humans. Med Sci Sports Exerc.

[13] Loo DDF, Zeuthen T, Chandy G, Wright EM (1996). Cotransport of water by the Na+/glucose cotransporter. Proc Natl Acad Sci USA.

[14] Pomeranz JL, Wilde P, Huang Y (2018). Legal and administrative feasibility of a federal junk food and sugar-sweetened beverage tax to improve diet. Am J Public Health.

[15] Minehan MR, Riley MD, Burke LM (2002). Effect of flavor and awareness of kilojoule content of drinks on preference and fluid balance in team sports. Int J Sport Nutr Exerc Metab.

[16] Ma J, Bellon M, Wishart JM (2009). Effect of the artificial sweetener, sucralose, on gastric emptying and incretin hormone release in healthy subjects. Am J Physiol Gastrointest Liver Physiol.

[17] Adibi SA (1980). Role of small intestine in digestion of protein to amino acids and peptides for transport to portal circulation. Curr Concepts Nutr.

[18] Bröer S, Fairweather SJ (2019). Amino acid transport across the mammalian intestine. Compr Physiol.

[19] Mailliard ME, Stevens BR, Mann GE (1995). Amino acid transport by small intestinal, hepatic, and pancreatic epithelia. Gastroenterology.

[20] Mahalanabis D, Patra FC (1983). In search of a super oral rehydration solution: can optimum use of organic solute-mediated sodium absorption lead to the development of an absorption promoting drug?. J Diarrhoeal Dis Res.

[21] Gauthier-Coles G, Vennitti J, Zhang Z (2021). Quantitative modelling of amino acid transport and homeostasis in mammalian cells. Nat Commun.

[22] Durnin B, Womersley J (1974). Body fat assessed from total body density and its estimation from skinfold thickness: measurements on 481 men and women aged from 16 to 72 years. Br J Nutr.

[23] Caballero-Plasencia AM, Valenzuela-Barranco M, Martín-Ruiz JL (1999). Are there changes in gastric emptying during the menstrual cycle?. Scand J Gastroenterol.

[24] Rodriguez-Giustiniani P, Galloway S (2019). Influence of peak menstrual cycle hormonal changes on restoration of fluid balance after induced dehydration. Int J Sport Nutr Exerc Metab.

[25] Minshull C, James L (2013). The effects of hypohydration and fatigue on neuromuscular activation performance. Appl Physiol Nutr Metab.

[26] Corney RA, Horina A, Sunderland C, James LJ (2015). Effect of hydration status and fluid availability on ad-libitum energy intake of a semi-solid breakfast. Appetite.

[27] May ME, Hill JO (1990). Energy content of diets of variable amino acid composition. Am J Clin Nutr.

[28] Dill DB, Costill DL (1974). Calculation of percentage changes in volumes of blood, plasma, and red cells in dehydration. J Appl Physiol.

[29] Narang BJ, Atkinson G, Gonzalez JT, Betts JA (2020). A tool to explore discrete-time data: the time series response analyser. Int J Sport Nutr Exerc Metab.

[30] Hill RJ, Bluck LJC, Davies PSW (2008). The hydration ability of three commercially available sports drinks and water. J Sci Med Sport.

[31] Fordtran JS (1975). Stimulation of active and passive sodium absorption by sugars in the human jejunum. J Clin Invest.

[32] Fordtran JS, Rector FC, Carter NW (1968). The mechanisms of sodium absorption in the human small intestine. J Clin Invest.

[33] Patra FC, Mahalanabis D, Jalan KN (1982). Stimulation of sodium and water absorption by sucrose in the rat small intestine. Acta Paediatr Scand.

[34] Gisolfi CV, Summers RW, Schedl HP (1990). Human intestinal water absorption: direct vs. indirect measurements. Am J Physiol.

[35] Evans GH, Shirreffs SM, Maughan RJ (2011). The effects of repeated ingestion of high and low glucose-electrolyte solutions on gastric emptying and blood 2H_2_O concentration after an overnight fast. Br J Nutr.

[36] Maughan RJ, Leiper JB, Vist GE (2004). Gastric emptying and fluid availability after ingestion of glucose and soy protein hydrolysate solutions in man. Exp Physiol.

[37] Vist GE, Maughan RJ (1994). Gastric emptying of ingested solutions in man: effect of beverage glucose concentration. Med Sci Sports Exerc.

[38] Knepper MA, Kwon T-H, Nielsen S (2015). Molecular physiology of water balance. N Engl J Med.

[39] Evans GH, James LJ, Shirreffs SM, Maughan RJ (2017). Optimizing the restoration and maintenance of fluid balance after exercise-induced dehydration. J Appl Physiol.

[40] Cheuvront SN, Kenefick RW (2016). Am I drinking enough? Yes, no, and maybe. J Am Coll Nutr.

[41] Evans GH, Shirreffs SM, Maughan RJ (2009). Postexercise rehydration in man: the effects of osmolality and carbohydrate content of ingested drinks. Nutrition.

[42] Clarke MM, Stanhewicz AE, Wolf ST (2019). A randomized trial to assess beverage hydration index in healthy older adults. Am J Clin Nutr.

[43] Maughan RJ, Watson P, Cordery PAA (2016). A randomized trial to assess the potential of different beverages to affect hydration status: development of a beverage hydration index. Am J Clin Nutr.

[44] Maughan RJ, Watson P, Cordery PAA (2019). Sucrose and sodium but not caffeine content influence the retention of beverages in humans under euhydrated conditions. Int J Sport Nutr Exerc Metab.

[45] Crowe MJ, Forsling ML, Rolls BJ (1987). Altered water excretion in healthy elderly men. Age Ageing.

