# Supplementary material for: Iterative assessment of a sports rehydration beverage containing a novel amino acid formula on water uptake kinetics
Source: Eur J Nutr. 2024 Feb 13;63(4):1125–37. doi: 10.1007/s00394-024-03325-x (PMC11139694; doi:10.1007/s00394-024-03325-x)
Supplement: Supplementary file 1 — Supplementary file1 (DOCX 982 KB) [file 394_2024_3325_MOESM1_ESM.docx]

**Supplementary Material**

**Iterative assessment of a sports rehydration beverage containing a novel amino acid formula on water uptake kinetics**

**European Journal of Nutrition**

Mark P. Funnell, Loris A. Juett, Kirsty M. Reynolds, Drusus A. Johnson, Ruth M. James, Stephen A. Mears, Samuel N. Cheuvront, Robert W. Kenefick & Lewis J. James

National Centre for Sport and Exercise Medicine, School of Sport, Exercise and Health Sciences, Loughborough University, Leicestershire, UK, LE11 3TU.

**Corresponding Author**

Dr. Lewis J. James

L.James@lboro.ac.uk

School of Sport, Exercise and Health Sciences

Loughborough University**Supplementary Material**

*Plasma Amino Acid Concentration*

Study 1: There were no significant interaction effects for plasma concentration for arginine, histidine, lysine, glutamine, cysteine, alanine, leucine, methionine, and tryptophan (*P*≥0.066). There were significant interaction effects for plasma concentration for aspartic acid, serine, valine, isoleucine, threonine, tyrosine, glutamic acid, asparagine, glycine, proline, phenylalanine, and total amino acids (*P*≤0.026). Significant *post-hoc* analyses are displayed in Supplementary Fig. 1.

Study 2: There were no significant interaction effects for plasma concentration for aspartic acid, arginine, histidine, lysine, glutamic acid, asparagine, cysteine, alanine, methionine, and tryptophan (*P*≥0.054). There were significant interaction effects for plasma concentration for serine, valine, isoleucine, threonine, tyrosine, glutamine, glycine, proline, leucine, phenylalanine, and total amino acids (*P*≤0.048). Significant *post-hoc* analyses are displayed in Supplementary Fig. 2.

**
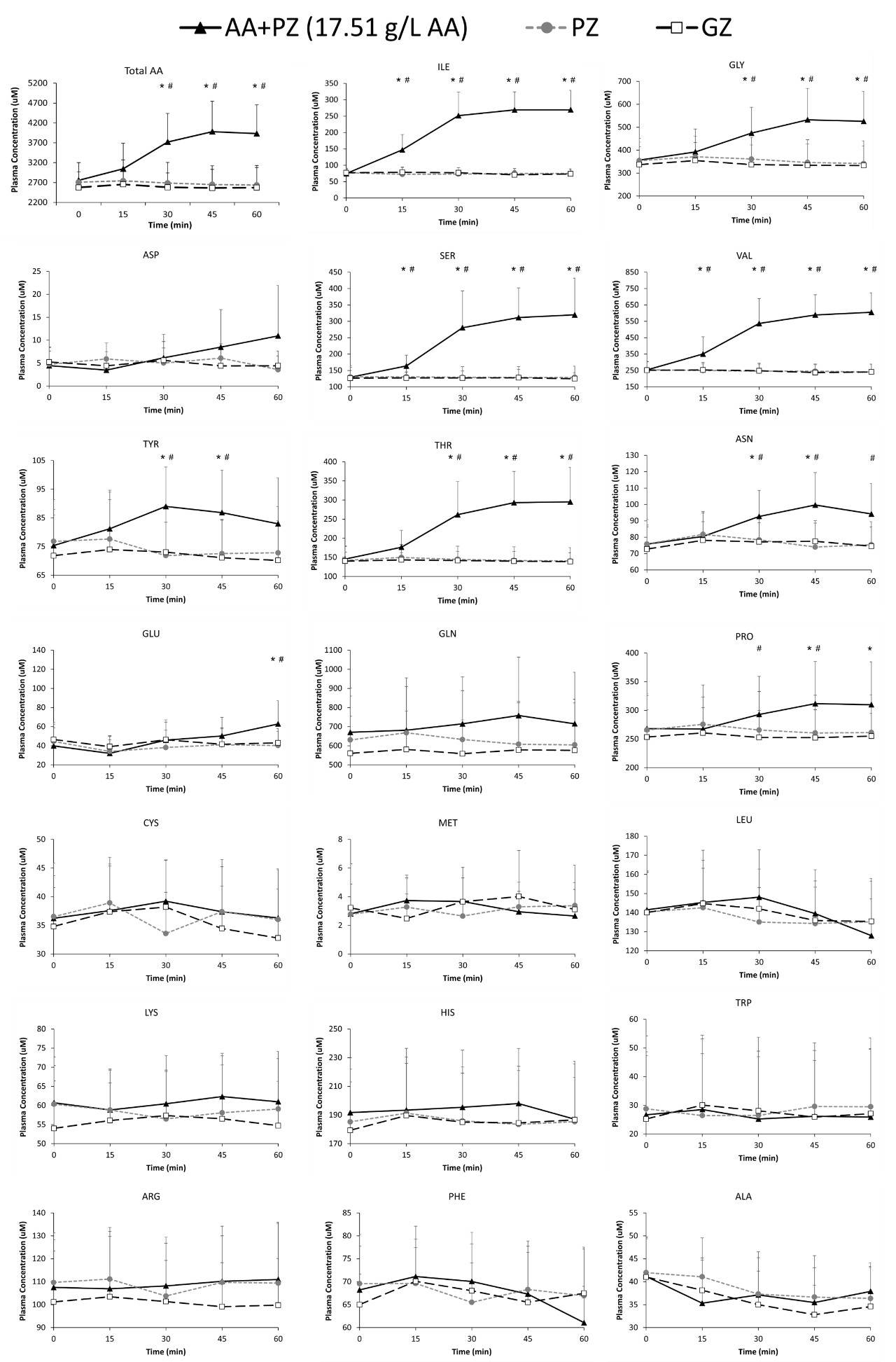
Supplementary Fig. 1.** Total and individual plasma amino acid concentration (µM) over time after ingesting the three experimental beverages for Study 1. * = AA+PZ significantly different to PZ. # = AA+PZ significnatly different to GZ. AA+PZ = a sugar-free rehydration beverage (PZ) containing 17.51 g/L of a novel amino acid formula. PZ = Powerade Zero™. GZ = Gatorade Zero™. **
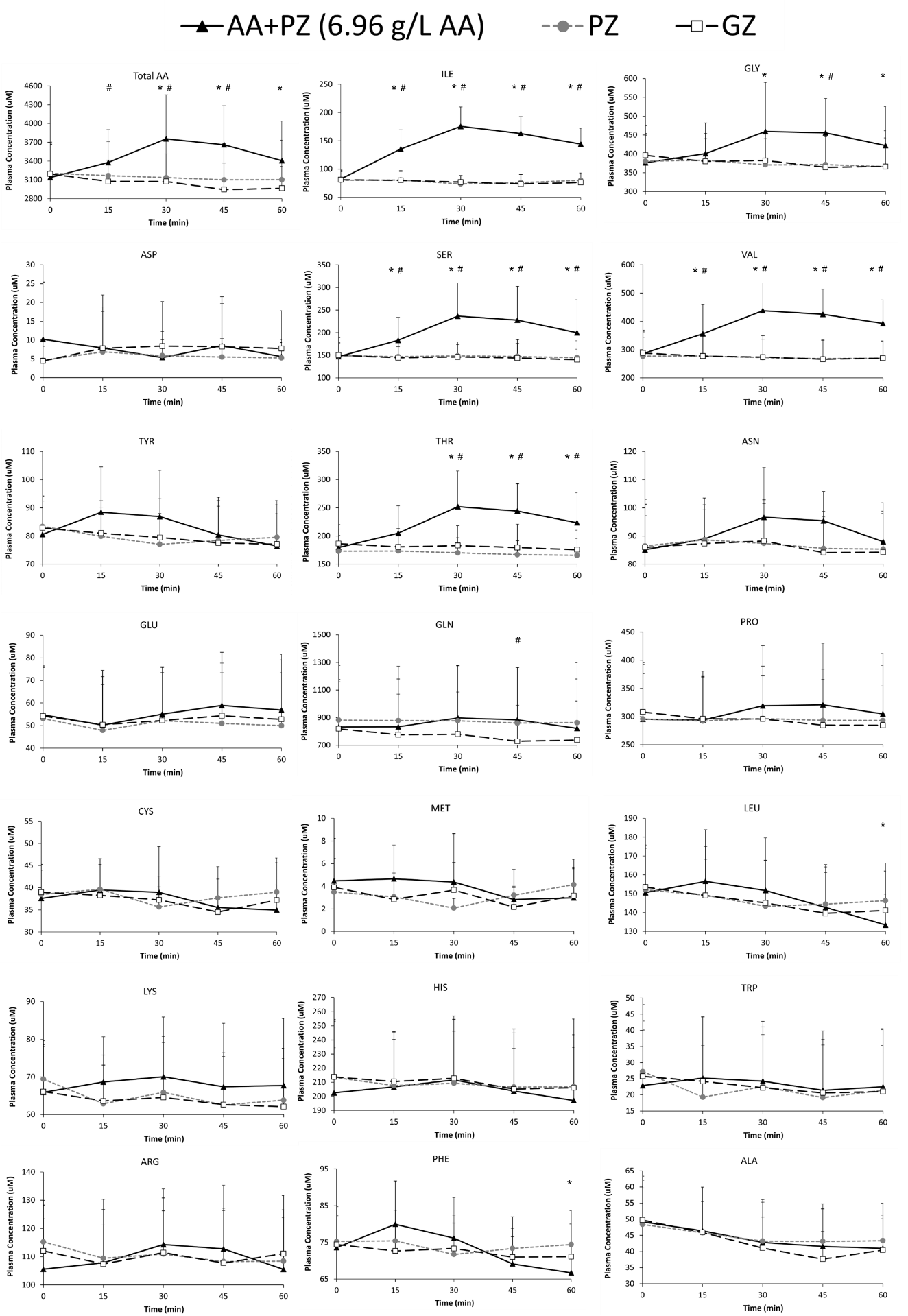
Supplementary Fig. 2.** Total and individual plasma amino acid concentration (µM) over time after ingesting the three experimental beverages for Study 2. * = AA+PZ significantly different to PZ. # = AA+PZ significnatly different to GZ. AA+PZ = a sugar-free rehydration beverage (PZ) containing 6.96 g/L of a novel amino acid formula. PZ = Powerade Zero™. GZ = Gatorade Zero™.

*Plasma Glucose*

Study 1: There were main effects for time and trial (both *P*<0.001), but no trial by time interaction effect (*P*=0.064) for plasma glucose, however, mean plasma glucose altered by <0.4 mmol/L across all time points and trials (Supplementary Fig. 3A).

Study 2: There was a main effect of time for plasma glucose (*P*<0.001), but there were no trial (*P*=0.135) or trial by time interaction (*P*=0.541) effects for plasma glucose (Supplementary Fig. 3B).

**Supplementary Fig. 3.** Plasma glucose (mmol/L) over time after ingesting the three experimental beverages for Study 1 **(A)** and Study 2 **(B)**. AA+PZ = a sugar-free rehydration beverage (PZ) containing differing amounts of a novel amino acid formula. PZ = Powerade Zero™. GZ = Gatorade Zero™.

*Plasma Lactate*

Study 1: There were main effects for time (*P*=0.004), trial (*P*<0.001), and a trial by time interaction effect (*P*<0.001) for plasma lactate, however, mean plasma lactate altered by <0.2 mmol/L across all time points and trials (Supplementary Fig. 4A).

Study 2: There were no time, trial, or trial by time interaction effects for plasma lactate (*P*≥0.247; Supplementary Fig. 4B).

**Supplementary Fig. 4.** Plasma lactate (mmol/L) over time after ingesting the three experimental beverages for Study 1 **(A)** and Study 2 **(B)**. AA+PZ = a sugar-free rehydration beverage (PZ) containing differing amounts of a novel amino acid formula. PZ = Powerade Zero™. GZ = Gatorade Zero™.

*Plasma Creatinine*

Study 1: For plasma creatinine, there was a main effect of time (*P*<0.001), but no trial (*P*=0.414) or trial by time interaction (*P*=0.217) effects (Supplementary Fig. 5A).

Study 2: For plasma creatinine, there was a main effect of time (*P*=0.001), but no trial (*P*=0.683) or trial by time interaction (*P*=0.381) effects (Supplementary Fig. 5B).

**Supplementary Fig. 5.** Plasma creatinine (mmol/L) over time after ingesting the three experimental beverages for Study 1 **(A)** and Study 2 **(B)**. AA+PZ = a sugar-free rehydration beverage (PZ) containing differing amounts of a novel amino acid formula. PZ = Powerade Zero™. GZ = Gatorade Zero™.

*Plasma Electrolytes*

Study 2: There was a main effect of time (*P*<0.001), but there were no trial (*P*=0.933) or trial by time interaction (*P*=0.404) effects for plasma sodium (Supplementary Fig. 6A). There was a main effect of time (*P*=0.008), but there were no trial (*P*=0.978) or trial by time interaction (*P*=0.397) effects for plasma potassium (Supplementary Fig. 6B).

**Supplementary Fig. 6.** Plasma **(A)** sodium (mmol/L) and **(B)** potassium (mmol/L) over time after ingesting the three experimental beverages for Study 2. AA+PZ = a sugar-free rehydration beverage (PZ) containing differing amounts of a novel amino acid formula. PZ = Powerade Zero™. GZ = Gatorade Zero™.

*Urine D_2_O Concentration*

Study 2: Post-trial (60 min) urine D_2_O enrichment was not different between trials (AA+PZ: 451 ± 137 δ‰ vs. VSMOW, PZ: 463 ± 130 δ‰ vs. VSMOW, GZ: 448 ± 155 δ‰ vs. VSMOW; *P*=0.708).
